# The Role of Single-Nucleotide Variants of *NOS1*, *NOS2,* and *NOS3* Genes in the Comorbidity of Arterial Hypertension and Tension-Type Headache

**DOI:** 10.3390/molecules26061556

**Published:** 2021-03-12

**Authors:** Natalia A. Shnayder, Marina M. Petrova, Polina V. Moskaleva, Pavel A. Shesternya, Elena A. Pozhilenkova, Regina F. Nasyrova

**Affiliations:** 1 V. F. Voino-Yasenetsky Krasnoyarsk State Medical University, 660022 Krasnoyarsk, Russia; stk99@yandex.ru (M.M.P.); sci-prorector@krasgmu.ru (P.A.S.); elena.a.pozhilenkova@gmail.com (E.A.P.); 2 V.M. Bekhterev National Research Medical Center for Psychiatry and Neurology, 199034 Saint-Petersburg, Russia

**Keywords:** nitric oxide, nitric oxide synthase, *single* nucleotide variants, polymorphisms, genes, *NOS1*, *NOS2*, *NOS3*, arterial hypertension, tension-type headache

## Abstract

Patients with tension-type headache (TTH) have an increased risk of developing arterial hypertension (AH), while hypertensive subjects do seem to have an increased risk of TTH. We searched for full-text English publications in databases using keywords and combined word searches over the past 15 years. In addition, earlier publications of historical interest were included in the review. In our review, we summed up the single nucleotide variants (SNVs) of Nitric Oxide Synthases (*NOSs*) genes involved in the development of essential AH and TTH. The results of studies we discussed in this review are contradictory. This might be due to different designs of the studies, small sample sizes in some of them, as well as different social and geographical characteristics. However, the contribution of genetic and environmental factors remains understudied. This makes the issue interesting for researchers, as understanding these mechanisms can contribute to a search for new approaches to pathogenetic and disease-modifying treatment of the AH and TTH phenotype. New drugs against AH and TTH can be based on inhibition of nitric oxide (NO) production, blockade of steps in the NO-cGMP pathway, or NO scavenging. Indeed, selective neuronal NOS (n-NOS) and inducible NOS (i-NOS) inhibitors are already in early clinical development.

## 1. Introduction

Arterial hypertension (AH) is a prevalent condition worldwide and is the key risk factor for non-fatal and fatal cardiovascular complications [1]. Tension-type headache (TTH) is the most common type of primary headache and is considered a common everyday headache [2]. Many studies support the hypothesis that TTH patients have an increased risk of developing AH, while hypertensive subjects do seem to have an increased risk of TTH. The relationship between AH and TTH is potentially of great pathophysiological and clinical interest, but it is poorly understood [3]. This allows us to hypothesize the presence of the AH and TTH phenotype. 

The pathophysiological patterns are significantly different in the setting of chronic pain, in which the adaptive relationship between blood pressure and pain sensitivity is reversed. The connection between acute or chronic pain and cardiovascular changes is supported observationally [4], but some of this indirect evidence is confirmed by experimental animal models and human studies. AH and TTH may share common mechanisms like endothelial dysfunction, deficiency of autonomic cardiovascular regulation, and renin angiotensin system involvement. 

Nitric oxide (NO) is an important autocrine and paracrine signaling molecule that plays a crucial role in cardiovascular physiology and pathology regulation. NO is an important molecule involved in regulation of cerebral and extra cerebral cranial blood flow and arterial diameters [5]. Reduced bioavailability of NO in the endothelium is an important precursor for impaired vasodilation and AH. Furthermore, NO is involved in nociceptive processing. A NO-induced biphasic response with an immediate and a delayed headache is common to chronic TTH in humans [6].

**Figure 1:**
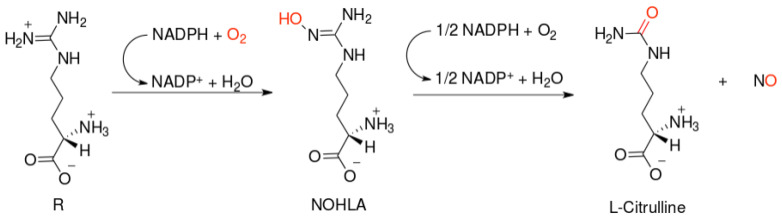
Scheme of nitric oxide (NO) formation from L-arginine in humans.

NO-synthases (NOSs) are expressed in three isoforms, including neuronal NOS (nNOS, *NOS1*), inducible NOS (iNOS, *NOS2*), and endothelial NOS (eNOS, *NOS3*) [7]. All NOS isoforms can catalyze the conversion of l-arginine to L-citrulline and NO (Figure 1). The active NOSs form a homodimer and convert the amino acid L-arginine to L-citrulline and NO. The NOS monomer contains a C-terminal reductase domain and a N-terminal oxygenase domain, which are linked by the calmodulin (CaM) binding region. The N-terminal oxidase domain contains the heme, tetrahydrobiopterin cofactors (BH4), and the binding site for the substrate arginine. The oxidase domain is the active site of NO synthesis. Production of NO requires O_2_ as an electron acceptor. NO diffuses freely across the plasma membrane. Therefore, NO can be transported to effector proteins in the adjacent cells and exert its effects (e.g., endothelial NO targets soluble guanylate cyclase (sGC) in smooth muscle to accomplish vasodilation) [8].

*NOS1* and *NOS3* are commonly associated with low levels of NO production, which mediates intracellular signaling processes (*NOS1*) and vascular homeostasis (*NOS3*). In addition to NO production, eNOS can function in an uncoupled manner and produce ROS when the available stores of BH4 are removed or oxidized, l-arginine is depleted, or the eNOS inhibitor asymmetric dimethyl-l-arginine is overexpressed [9,10]. nNOS and eNOS are most commonly found in non-immunological cells (e.g., neurons, muscle, endothelium). As their NO output is relatively low, these isoforms are considered less immunologically important than their inducible counterpart, iNOS [7].

NO and NOSs play an important role in the pathogenesis and treatment of both AH and TTH. In particular, NOSs affect the sensitivity of the brain to stress and changes in systemic and local hemodynamics [11]. In addition, a NO-synthesis change in endothelial cells of peripheral and cerebral vessels is one of the identified causes of NO-dependent vasospasm (impaired NO-dependent vasodilation) in patients with AH [12] and TTH [13], as well as chronic pain syndrome [13,14] and changes in the response to antihypertensive drugs [15] and analgesics [16,17]. However, the functional activity of enzymes of the NOS family depends on the carriage of wild, highly functional, low functional, and non-functional single nucleotide variants (SNVs) of *NOS1*, *NOS2*, and *NOS3* genes encoding the enzyme isoforms.

The aim of the review is to study the role of SNVs of the *NOS1*, *NOS2,* and *NOS3* genes in the comorbidity of arterial hypertension and tension-type headache.

## 2. Results

The NOS family proteins are encoded by the genes *NOS1* (also known as: *N-NOS*, *NC-NOS*, *nNOS*, *bNOS*, *IHPS1*), *NOS2* (also known as: *I-NOS*, *NOS2A*, *iNOS*, *HEP-NOS*), and *NOS3* (also known as: *eNOS*, *ECNOS*) [18]. SNVs of genes encoding NOSs can affect their expression level and/or activity in organs and tissues. Experimental evidence suggests that nNOS (*NOS1*), iNOS (*NOS2*), and eNOS (*NOS3*) have important effects on cardiovascular function and pain, but their cumulative effect on the AH and TTH phenotype in humans is unknown. Therefore, the phenotype requires a better treatment. Association studies related to the role of SNVs of *NOS1*, *NOS2,* and *NOS3* genes in the development of AH and TTH phenotype are of scientific and clinical interest.

### 2.1. NOS1 Gene

The *NOS1* gene is localized on chromosome 12q24.22 (exon count: 33) [18]. There are biased expressions of *NOS1* in brain (RPKM 1.1), kidney (RPKM 1.0), and 11 other tissues [19]. This protein is ubiquitously expressed, especially in skeletal muscle. Multiple transcript variants that differ in the 5′ UTR have been described for the *NOS1* gene, but the full-length nature of these transcripts is not known. Additionally, alternatively spliced transcript variants encoding different isoforms (some testis-specific) have been found for this gene [18].

Levinsson A. et al. (2014) found that the carriage of the T allele rs3782218 of the *NOS1* gene reduces the risk of developing AH (odds ratio (OR) = 0.81, 95% confidential interval (CI) = 0.67–0.97, *p* = 0.02), and the carriage of the A allele rs7314935, on the contrary, increases this risk (OR = 2.15, CI = 1.06–4.37, *p* = 0.03) [20]. The role SNVs in headache development (Alaşehirli B. et al. (2013) [21] and García-Martín E. et al. (2015) [22]) was studied, but no associations were found in patients with migraine.

### 2.2. NOS2 Gene

The *NOS2* gene is localized on chromosome 17q11.2 (exon count: 27) [18]. There are biased expressions of *NOS2* in the small intestine (RPKM 10.3), appendix (RPKM 7.9), and five other tissues [19]. The inducible NOS (iNOS) function produced by the *NOS2* gene is activated to combat environmental factors.

Fu L. et al. (2009) investigated the association between SNV rs2779249 (−1026C/A) of the *NOS2* promoter and susceptibility to AH in Chinese Han [23]. Significant differences were found in the genotype and allele frequencies of the *NOS2* rs2779249 (*p* < 0.05); the genotype CC was associated with AH after adjusting for environmental risk factors through nonconditional logistic regression analysis (adjusted OR = 2.90; CI = 2.14–3.93). Transmission disequilibrium test analysis demonstrated that allele C was preferentially transmitted within pedigree (combined Z score 2.257, *p* < 0.05). The *NOS2* rs2779249 was identified by a construct reporter assay as a functional variant, and the transcriptional activity of the promoter with allele C was 4.73-fold lower than that with allele A. Furthermore, a electrophoresis mobility shift assay showed that rs2779249 changed the Y in Yang 1 (YY1)-binding pattern in vitro, whereas chromatin immunoprecipitation showed that transcription factor YY1 was bound to the variant 1026C (rs2779249) *in vivo*. Lipopolysaccharide, an inflammatory stimulating factor, could induce YY1 to augment DNA-binding affinity; it could also be involved in the inhibited transcriptional activity of the *NOS2* promoter with allele C. The authors thus conclude that *NOS2* rs2779249 with a change in YY1-binding affinity is associated with AH due to the effect of inflammatory-stimulating factors [23].

The risk of essential AH was assessed in carriers of the *NOS2* gene variants rs1800482 (−954G>C) and rs3730017 (C>T). In subjects carrying allele C (rs1800482), the risk of essential AH developing was 1.7 times higher (OR = 1.712, 95%CI 1.07–2.74), while the presence of the T allele (rs3730017) had a protective effect (OR = 0.304, 95%CI 0.192–0.482). In patients with essential AH, the presence of the C allele (rs1800482) was associated with a higher content of NO metabolites in the blood plasma [24]. Furthermore, Topchieva L.V. et al. (2019) determined the influence of the C allele on the *VCAM1* and *ICAM1* gene expression in patients with essential AH. It was hypothesized that this polymorphic site in the *NOS2* gene can be involved in the development of endothelial dysfunction and essential AH through modulation of the NO level in inflammation [24].

Less is known on whether the two SNVs in the *NOS2* gene (rs2779249 (1026C>A)), rs2297518 (2087G>A)) affect susceptibility to AH. Nikkari S.T. et al. (2015), in the Tampere adult population cardiovascular risk study (TAMRISK), established the association between these SNVs and AH diagnosed in a Finnish cohort [25]. Data analysis was done by logit regression. At the age of 50, the SNP rs2779249 is associated significantly with AH (*p* = 0.009); specifically, subjects carrying the A-allele had higher risk of AH compared to those carrying the CC genotype (OR = 1.47; CI = 1.08–2.01; *p* = 0.015). In addition, a 15-year follow-up period (35, 40, and 45 years) of the same individuals showed that carriers of the A-allele had AH more often in all of the studied age groups. The highest risk of developing AH was observed among 35-year-old subjects (OR = 3.83; CI = 1.20–12.27; *p* = 0.024). Those carrying variant A also had significantly higher readings of both systolic (*p* = 0.047) and diastolic (*p* = 0.048) blood pressure during the follow-up. No significant associations between rs2297518 variant alone and AH were found. However, haplotype analysis of rs2779249 and rs2297518 revealed that individuals having haplotype H3, which combines both A alleles (CA-GA, 19.7% of individuals), were more commonly found in the hypertensive group than in the normotensive group (OR = 2.01; CI = 1.29–3.12; *p* = 0.002). In conclusion, there was a significant association between the *NOS2* genetic variant (rs2779249) and AH in the genetically homogenous Finnish population. Those who carried the rare A-allele of the gene already had higher risk for AH at the age of 35 years [25].

Zhai Z. et al. (2018) studied the association between SNVs rs2779249 and rs2297518 of the *NOS2* gene and AH in a Han Chinese cohort [26]. Logit regression analyses were performed with different genetic models (additive, dominant, and recessive) adjusting for confounding risk covariates, including age, sex, body mass index, total cholesterol, triglycerides, high-density lipoprotein cholesterol, low-density lipoprotein cholesterol, smoking, drinking, and family history of hypertension. The OR was 1.27 (CI = 1.12–1.44) in the additive model, 1.31 (CI = 1.09–1.59) in the dominant, and 1.68 (CI = 1.28–2.19) in the recessive model of rs2779249; the OR was 1.26 (CI = 1.06–1.50) in the additive model and 1.46 (CI = 1.13–1.89) in the dominant model of rs2297518. The current study provides evidence that *NOS2* is strongly associated with AH [26].

While genetic SNVs affect NOS2 expression, it is not known whether *NOS2* gene polymorphisms affect the susceptibility to AH and the responses to antihypertensive therapy. Oliveira-Paula G.H. et al. (2013) studied whether *NOS2* polymorphisms ((CCTTT)(n), rs2779249, andrs2297518) and haplotypes are associated with AH and responsiveness to drug therapy [27]. The authors studied 115 well-controlled hypertensive patients (HTN), 82 hypertensive patients resistant to optimized antihypertensive therapy (RHTN), and 113 normotensive healthy subjects (NT). The PHASE 2.1 software was used to estimate the haplotype frequencies in each group. Variant genotypes (GA + AA) for the rs2297518 polymorphism were more commonly found in hypertensive patients (HTN + RHTN) than in normotensives (*p* = 0.016; OR = 2.05). Authors found no associations between genotypes and responsiveness to therapy (*p* > 0.05). The S-C-A haplotype was more commonly found in hypertensive patients (HTN + RHTN) than in NT (*p* = 0.014; OR = 6.07). Interestingly, this haplotype was more commonly found in the HTN group than in the RHTN group (*p* = 0.012; OR = 0.14). The rs2297518 polymorphism in the *NOS2* gene affects the susceptibility to AH. Moreover, while the S-C-A haplotype is associated with AH, it is also associated with responsiveness to antihypertensive therapy [27].

*NOS2* SNVs associative studies showed the relationship of rs2779249 and rs2297518 in haplotypes with an increased risk of primary headaches [28,29].

### 2.3. NOS3 Gene

The *NOS3* gene is localized on chromosome 7q36.1 (exon count: 28) [18]. There are biased expressions of *NOS3* in spleen (RPKM 28.4), placenta (RPKM 4.0), fat (RPKM 3.1), heart (RPKM 2.0), and nine other tissues [19]. Vascular endothelial cells produce NO, which contributes to the regulation of blood pressure and regional blood flow. The human endothelial *NOS* (*NOS3*) gene is highly polymorphic. The SNVs of the *NOS3* gene are associated with AH development.

Yasujima M. et al. (1998) [30] examined the possible involvement of the *NOS3* gene in the genetic basis for AH. The authors identified genotypes for two SNVs of the *NOS3* gene in hypertensive and normotensive populations of northern Japan. The specific genotypes for rs1799983 (Glu298Asp or +894G>T) missense variant and variable numbers of tandem repeats in intron 4 (NOS3 VNTR 4b/4a) were isolated using allele-specific gene amplification and restriction fragment length polymorphisms. The 298Asp variant was significantly correlated to AH in these groups (OR = 1.8; CI = 1.1–3.2). The allelic frequencies of 298Asp for Glu298 in patients with AH were significantly higher than those in normotensive subjects (0.136 vs. 0.083, *p* < 0.05). However, there was no disequilibrium of *NOS3* 4b/4a between these two groups. The results suggest that rs1799983 is a genetic susceptibility factor for AH [30]. Molecular studies have indicated that intact *NOS3* Asp298 has equivalent enzymatic activity in relation to *NOS3* Glu298, but undergoes selective proteolysis in native cells and tissues in such a way that the steady-state level of active *NOS3* may be reduced in carriers of this allele. Carriers of *NOS3* Asp298, particularly if exposed to adverse environmental influences on endothelial function, may be at increased risk of developing cerebrovascular disease, including essential AH and pre-eclampsia [31].

Moe K.T. et al. (2006) conducted the association analysis of *NOS3* gene polymorphisms with essential AH in a Singapore population [32]. The specific genotypes for rs1799983 in exon 7, VNTR in intron 4 (*NOS3* 4A/B/C) and rs2070744 (−786T>C) in the promoter were isolated using allele-specific gene amplification and restriction fragment length polymorphisms to examine the association of genotype and allelic frequency in both groups. Logit regression analysis was also used to detect the association between genotypes and AH. Five genotypes of intron 4 VNTR (AA, AB, BB, AC and BC) were observed. The intron 4 B/B genotype was significantly associated with the AH group (*p* = 0.035), but there was no disequilibrium of rs1799983 and rs2070744 between the two groups (*p* = 0.419 and *p* = 0.227), respectively. The overall distribution of allelic frequency differed significantly between the two groups, with the four-repeat allele (4A) of intron 4 more frequent in the normotensive group than the AH group (*p* = 0.019). Logit regression analysis showed that the intron 4 B/B genotype was significantly associated with systolic blood pressure of individuals with body mass index (BMI) greater than 25 kg/m^2^ (*p* = 0.04). In conclusion, the *NOS3* 4 B/B genotype is a genetic susceptibility factor for essential AH in a Singapore population [32]. 

The SNV rs1799983 of the *NOS3* gene has been suggested to be responsible for reduced NO synthesis and essential AH development. Nassereddine S et al. (2018) evaluated the potential association of SNV rs1799983 of the *NOS3* gene with AH susceptibility among a sample of Moroccan patients [33]. The results showed a positive correlation between rs1799983 *NOS3* distribution and alcohol and obesity risk factors (*p* = 0.009 and 0.02, respectively). Patients with elevated cardiovascular risk (CVR) had a higher frequency of homozygous mutant genotype TT (62.2%) and T mutant allele (77.8%), compared to median and low CVR groups. The SNV rs1799983 of *NOS3* distribution was significantly associated with a high risk of AH occurrence under the GT and TT genotypes (OR = 20.2, CI = 7.7–52.4, *p* < 0.0001; OR = 332.5, CI = 98.2–1125.4, *p* < 0.0001; respectively), and the three genotypic transmission models (Dominant: OR = 43.2, CI = 17.9–104.09, *p* < 0.0001; Recessive: OR = 47.7, CI = 18.6–122.3, *p* < 0.0001; Additive: OR = 14.02, CI = 9.6–20.45, *p* < 0.0001). This study suggests a strong association of rs1799983 of the *NOS3* gene with AH susceptibility in Morocco. Studies trying to identify contributing genes may be very useful for recognizing vulnerable individuals and classifying patients in subgroups with definite genetic and pathogenic mechanisms to achieve better prevention and therapy of AH [33]. In addition, a prognostic role of rs1799983 of the *NOS3* gene in AH development was shown by Zhang L.P. et al. (2006) [34] and Men C. et al. (2011) [35] in China. A meta-analysis by Li Y.Y. (2011) [36] included the results of studies of the association between rs1799983 of the *NOS3* gene and AH; electronic databases such as PubMed, Embase, Web of Science, China Biological Medicine Database (CBMD), and China National Knowledge Infrastructure (CNKI) were used. In the current meta-analysis, the T allele of SNV rs1799983 of the *NOS3* gene was suggested to be related to the increased risk of AH in the Chinese population, particularly in those of Han ethnicity [36]. On the contrary, Tsujita Y. et al. (2001) [37] suggested that these SNVs (rs1799983 and rs2070744) of the *NOS3* gene are unlikely to be major factors in AH susceptibility in the studied Japanese population.

An Italian study by Rossi G.T. et al. (2003) [38] showed that rs2070744 and its interaction with rs1799983 of the *NOS3* gene affects endothelium-dependent vasodilation in mild-to-moderate AH patients and healthy normotensive Caucasian subjects.

Gamil S. et al. (2017) investigated the association between these three SNVs (rs1799983, a VNTR in intron 4, and rs2070744) in the *NOS3* gene and AH development in Sudanese patients [39]. The rs2070744 polymorphism in *NOS3* was found to be associated with AH in the Sudanese population as the patients group had higher frequency of the CC genotype compared to the controls (6.6% vs. 6.1%, *p* = 0.02). Considering the dominant inheritance model, the frequency of TC + CC genotypes in patients was significantly higher than that in the control subjects (52.6% vs. 34.1%, respectively; *p* < 0.01), with an OR (95% CI) of 2.14 (1.23–3.74). In addition, the C allele was more frequent in the patients than in the control group (29.6% vs. 20%, *p* = 0.03, OR = 1.84 (1.15–2.93)). The C allele of intron 4 VNTR was reported in >1% of the Sudanese population under study. The results of this study indicated that the rs2070744 polymorphism in *NOS3* may be a genetic susceptibility factor for essential AH in the Sudanese population. The C allele of intron 4 VNTR is not rare in the Sudanese population [39].

Salvi E. et al. (2013) [40] revealed an association between AH and rs3918226 in the *NOS3* gene promoter (minor/major allele, T/C allele). The authors aimed at substantiating these preliminary findings by target sequencing, cell experiments, and a population study in 2722 randomly recruited Europeans (53.0% women; mean age 40.1 years). Change in the blood pressure and incidence of AH in relation to rs3918226 were studied using multivariable-adjusted models. They sequenced the 140-kb genomic area encompassing the *NOS3* gene. Sequencing confirmed rs3918226, a binding site of twenty-six transcription factors, as the SNV most closely associated with AH. In T, as compared to C transfected cells, *NOS3* promoter activity was from 20% to 40% (*p* < 0.01) lower. In the population, systolic/diastolic blood pressure increased over 7.6 years (median) by 9.7/6.8 mm Hg in 28 TT homozygotes and by 3.8/1.9 mm Hg in C allele carriers (*p* ≤ 0.0004). The blood pressure rise was 5.9 mm Hg systolic (CI = 0.6–11.1; *p* = 0.028) and 4.8 mm Hg diastolic (CI = 1.5–8.2; *p* = 0.0046) greater in TT homozygotes, with no differences between the CT and CC genotypes (*p* ≥ 0.90). Among 2013 participants normotensive at baseline, 692 (34.4%) developed AH. The hazard ratio and attributable risk associated with TT homozygosity were 2.04 (CI = 1.24–3.37; *p* = 0.0054) and 51.0%, respectively. In conclusion, rs3918226 in the *NOS3* promoter tags an AH susceptibility locus, TT homozygosity being associated with lesser transcription and higher risk of AH [40].

The incidence of AH is increasing and is more common in men than in women. Up to date, the MMP3 5A/6A polymorphism has been associated with artery stiffening and elevated blood pressure, whereas results considering association of rs1799983 polymorphism of the *NOS3* gene with AH are controversial. Djurić T. et al. (2005) [41] analyzed the possible association of rs1799983 and MMP3 5A/6A polymorphisms of the *NOS3* gene with AH in a Serbian population. The study included hypertensive and normotensive subjects. There was a significantly higher (*p* < 0.05) prevalence of 5A/5A genotype in hypertensive females compared to normotensive ones (19.30% vs. 10.84%). This prevalence was even more pronounced in females 50 years old and above, according to its recessive effect. In young males (<40 years), the study revealed a 3.7-fold increased risk for AH associated with allele 6A (*p* < 0.01), and 8.1-fold with genotype 6A/6A (*p* = 0.01) according to the recessive model. The authors found no association of rs1799983 of *NOS3* with AH. These results indicated that there were gender- and age-specific differences in the association of the MMP3 5A/6A polymorphism with AH in the Serbian population [41]. In addition, association of the rs1799983 polymorphism of the *NOS3* gene with the development of pre-eclampsia in women was found [42].

Most hypertensive patients require two or more drugs to control arterial blood pressure effectively. Although *NOS3* haplotypes have been associated with AH, it is unknown whether *NOS3* genotypes/haplotypes are associated with resistance to antihypertensive therapy. Sandrim V.C. et al. (2006) [43] studied the distribution of three SNVs of the *NOS3* gene: SNVs in the promoter region (rs2070744, rs1799983, and VNTR in intron 4 (b/a)). Genotypes were determined for 111 normotensive controls (NT), 116 hypertensive individuals who were well controlled (HT), and 100 hypertensive individuals who were resistant to conventional antihypertensive therapy (RHT). The authors also compared the distribution of *NOS3* haplotypes in the three groups of subjects. No differences were found in genotype or allele distribution among the three groups (all *p* > 0.05). Conversely, the ‘C Glu b’ haplotype was more commonly found in the NT than in the HT or RHT groups (21 versus 8 and 7%, respectively; both *p* < 0.00625). In addition, the ‘C Asp b’ haplotype was more commonly found in the HT and RHT groups than in the NT group (22 and 20%, respectively, versus 8%; both *p* < 0.00625). The distribution of *NOS3* haplotypes was not significantly different in the HT and RHT groups (*p* > 0.05). Whereas the findings suggest that (1) there is a protective effect for the ‘C Glu b’ haplotype against AH, (2) the ‘C Asp b’ haplotype increases AH susceptibility, and (3) *NOS3* haplotypes are not associated with resistance to antihypertensive therapy [43].

Quite a few studies have considered associations of *NOS3* gene SNVs with headache. However, these works used migraine patients: Borroni B. et al. (2006) [44], Eröz R. et al. (2014) [45] showed associations with rs1799983; Gonçalves F.M. et al. (2011 [46], 2012 [22]) showed associations with rs743506; Eröz R. et al. (2014) [45], Zakerjafari M. et al. (2016) [47] showed associations with rs2070744.

## 3. Discussion

### 3.1. Role of NO and NOSs in Pathogenesis and Treatment of AH

AH is characterized by endothelial dysfunction, vasoconstriction and microvascular rarefaction. All of these mechanisms are interconnected by cause and effect relationships. Moreover, NO is involved in each of these pathogenetic mechanisms.

Thus, NO is the main mediator of endothelial dysfunction underlying the development of AH. Endothelial dysfunction is the first step in the development of atherosclerosis; it is characterized by reduced biosynthesis and decreased bioavailability of NO [11]. NO is the active radical form of both oxygen (ROS) and nitrogen (RNS). These compounds can participate in free radical chain reactions or damage organic substrates. Chain reactions are one of the main reasons why free radicals can cause damage far from where they form. Any organ or system can be exposed to oxidative or nitrosative stress. However, the most susceptible to stress are the brain (high metabolic activity and low levels of endogenous antioxidants) and the circulatory system (fluctuations in oxygen and nitric oxide levels).

In AH, iNOS dysfunction is observed to a greater extent, since it is an isoform that catalyzes the formation of NO by endothelial cells. Li Q. et al. (2015) described the iNOS uncoupling mechanism. Tetrahydrobiopterin (BH4) is a key cofactor responsible for normal electron transfer from the reductase domain of one NOS2 monomer to the oxygenase domain of another monomer to form NO. When this cofactor is deficient, iNOS produces a superoxide anion instead of NO [48], which marks the induction of oxidative stress in the vessels. There are a number of studies that confirm this mechanism: with the use of an animal model [49,50] and in clinical trials [51,52,53].

Kelm M. et al. (1996) identified three causes of NO-dependent vasospasm (impaired NO-dependent vasodilation) in patients with AH: (1) reduced NO synthesis by endothelial cells due to impaired signal transduction and/or decreased NO synthase activity, (2) accelerated NO degradation in the vessel wall, and (3) structural disorders in the vessel wall leading to a general decrease in the dilator capacity of resistant arteries [12]. Endothelial cells release NO in response to shear stress and activation of various receptors. NO stimulates guanylyl cyclase to form 3′,5′-cyclic guanosine monophosphate, which leads to vasodilation of vascular smooth muscle cells. NO has a vasodilating and antiproliferative effect on smooth muscle cells and inhibits thrombocyte aggregation and leukocyte adhesion [11].

Increased NO biosynthesis enhances angiogenesis. Conversely, angiogenesis is impaired when NO levels are reduced (e.g., in *NOS2* knockout animal models). That is, conditionally, angiogenesis begins in response to hypoxia. Thus, signs of impaired angiogenesis and, as a result, microvascular rarefaction are revealed in AH [54]. It is important that the degree of AH does not affect angiogenesis, and microvascular rarefaction is also observed in normotensive people with a family history of hypertension. The genetic basis of these mechanisms has also been studied. Recent studies have confirmed that the pathway of L-arginine conversion to NO is impaired not only in people with AH, but also in people with normotension with a history of essential AH [55].

Vascular tone is regulated by a variety of autocrine and paracrine systems. The vascular renin-angiotensin system, kallikrein-kinin system, natriuretic peptide system, endothelin, mechanosensitive ion channels, prostanoids, catecholamines, and endothelial hyperpolarizing factor are involved [12]. The impact on these systems is already actively used in AH therapy. Interestingly, the effects of many cardiovascular drugs used in hypertension affect the NO system. Activating NO signaling or increasing NO bioavailability are key mechanisms contributing to the positive cardiovascular effects of drugs [56]. Angiotensin-converting enzyme inhibitors, calcium channel blockers, third-generation beta blockers, and statins significantly improve endothelial function and NO bioactivity [57]. Angiogenic growth factors (vascular endothelial growth factor (VEGF) and fibroblast growth factor (FGF)) activate NOS because they require NO to function. They do this through effector molecules and their effect on the RAAS receptors. For example, bradykinin and angiotensin II induce angiogenesis [54].

Based on this, new treatment plans targeting the NO system are currently being investigated and developed; they include NO donorship and NOS stimulants [57]. In 1996, Preik M. et al. (1996) showed the effectiveness of NO donorship (glycerol trinitrate, isosorbide dinitrate and sodium nitroprusside), and also noticed that it depended not on the drug class, but on the severity of AH [58]. These data form the basis of modern research. Rajapakse N.W. et al. (2019) suggested that the decrease in NO bioavailability makes a significant contribution to the development of resistant AH. This means that NO donorship may be the most effective treatment in this cohort of patients [59]. Today, these are pilot projects and developments, and the first clinical trials. However, they are promising and of great scientific and clinical interest.

It is assumed that drug action on the NO system, in addition to other links of therapy, is especially advisable in the case of resistant AH and comorbidity with other diseases (heart failure, diabetes mellitus, obesity). NO-mediated endothelial dysfunction in such patients is more pronounced. Consequently, today, when developing drugs for AH treatment, special attention is paid to the NO system. For example, nebivolol (a third generation β-blocker) is highly selective for β1-adrenergic receptors and causes vasodilation through interaction with the L-arginine/NO endothelial pathway. Although nebivolol lowers blood pressure to the same extent as conventional β-blockers and other types of antihypertensive drugs, it will have a positive effect on the vascular endothelium [60]. Nebivolol releases NO, thereby preventing the development of hypertension associated with chronic NO deficiency, and this effect appears to be dependent on inhibition of the renin-angiotensin system [15].

### 3.2. Role of NO and NOSs in Pathogenesis and Treatment of TTH

TTH is a highly prevalent disorder with a significant impact on society. Understanding the pathophysiology of TTH is paramount for development of effective treatments and prevention of chronic TTH [61]. Advances in basic pain and clinical research have improved our understanding of the TTH pathophysiologic mechanisms [13]. Pain perception studies such as measurement of muscle tenderness, pain detection thresholds, pain tolerance thresholds, pain response to suprathreshold stimulation, temporal summation and diffuse noxious inhibitory control have played a central role in elucidating the pathophysiology of TTH [61]. Increased excitability of the central nervous system (CNS) generated by repetitive and sustained pericranial myofascial input may be responsible for the transformation of episodic TTH into the chronic form [13]. Molecular mechanisms that underlie TTH have not yet been clarified. Studies in which TTH was induced by intravenous infusions of glyceryl trinitrate (an exogenous NO donor) and histamine (which liberates NO from vascular endothelium) suggest that NO is likely to be a responsible molecule. The release of NO from blood vessels, perivascular nerve endings or from CNS tissue is an important molecular trigger mechanism in spontaneous headache pain [14]. Sarchielli P. et al. (2001) assessed the variations in L-arginine/NO pathway activity and platelet cyclic guanosine 3′,5′-monophosphate (cGMP) levels in patients affected by chronic TTH. A reduction in platelet aggregation response was found. The reduction in platelet aggregation was coupled with increased NO and cGMP production. A significant increase in cytosolic Ca(2+) concentration was also detected compared to control individuals. This was accompanied by a reduced platelet content and collagen-induced secretion of serotonin and increased content of NO in patients with TTH. The above findings were more pronounced in patients with analgesic abuse. It can be hypothesized that the increased NOS activity shown in platelets of TTH patients reflects an analogous central up-regulation of NOS activity in the spinal horn/trigeminal nucleus and supraspinal structures; these structures are involved in the modulation of nociceptive input from myofascial cranial structures contributing to central sensitization. The increase in NOS activity seems to be associated with a hyposerotonergic status, particularly in patients with analgesic abuse. This can contribute to central sensitization in patients with TTH. The increase in platelet glutamate content in the same patients suggests the implication of the above excitatory amino acid in spinal and supraspinal structures involved in head pain induction and maintenance [62].

Tenderness of pericranial myofascial tissues and number of myofascial trigger points are considerably increased in patients with TTH. Mechanisms responsible for the increased myofascial pain sensitivity have been studied extensively. Peripheral activation or sensitization of myofascial nociceptors could be one of the causes for increased pain sensitivity, but firm evidence for a peripheral abnormality is still lacking. Peripheral mechanisms are most likely of major importance in episodic TTH [63]. Sensitization of pain pathways in CNS due to prolonged nociceptive stimuli from pericranial myofascial tissues seems to be responsible for TTH chronification. Neck muscle nociception mediated by NO may play a role in TTH pathophysiology [16]. The role of NO in the antinociceptive effect of indomethacin was assessed in the pain-induced functional impairment model in the rat (PIFIR model); the antinociceptive effect of indomethacin involves, at least in part, the NO-cyclic GMP pathway at the peripheral level [64]. Furthermore, the role of NO in the antinoceptive effect of other drugs used for TTH treatment (diclofenac [65]; ketorolac [66]; nimesulide [67]; celecoxib [68]; rofecoxib [69]; gabapentin [70]; melatonin [71]) has been shown.

Chronic TTH may be caused by prolonged painful input from pericranial myofacial tissues, such as tender points, which results in central sensitization (increased excitability of neurons in the CNS). Animal studies have shown that sensitization of pain pathways may be caused by or associated with activation of nNOS and generation of NO. Furthermore, it has been shown that NOS inhibitors reduce central sensitization in animal models of persistent pain [72].

nNOS is involved in the induction but not the maintenance of nerve growth factor (NGF) caused by facilitation of nociception in the brainstem. The results from an experimental animal model may support the idea of nNOS and eNOS as potential targets for pharmacological treatment of TTH [16]. Infusion of α,β-methylene ATP (α,β-meATP) into murine neck muscle facilitates brainstem nociception. Unspecific NOSs inhibition prevents and reverses this sensitization. It is unclear whether nNOS, iNOS or eNOS isoenzymes are involved in this α,β-meATP effect. Ristic D. et al. (2010) provided evidence that nNOS plays a major role in induction and eNOS in maintenance of facilitation in neck muscle nociception. Divergent roles of NOS isoenzymes may promote research on target-specific treatments for headache and neck muscle pain [17].

The role of nNOS and iNOS in central sensitization induced by an intradermal capsaicin injection was investigated by Wu J. et al. (2001) [73]. Obtained results by Budziñski M. et al. (2000) suggest that NO derived from iNOS plays an inhibitory role in carrageenan-produced hyperalgesia in rat [74].

The analgesic effect of the NOS inhibitor L-N(G) methyl arginine hydrochloride was investigated. This drug significantly reduced headache and myofascial factors in patients with chronic TTH. These studies show that NO plays a crucial role in the pathophysiology of TTH. The analgesic effect of NOS inhibition in patients with chronic TTH is probably due to a reduction in central sensitization at the level of the spinal dorsal horn, trigeminal nucleus or both. Furthermore, NOS inhibition may become a novel principle in the future treatment of chronic TTH [72].

### 3.3. Role of SNVs of NOSs Genes in the AH and TTH Phenotype and Comorbidites

**Figure 2:**
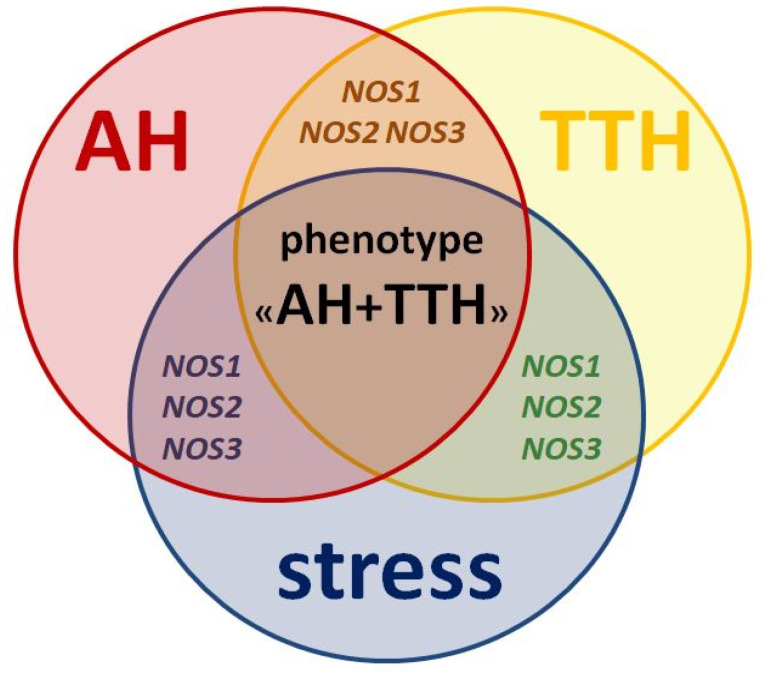
Similarities in pathogenesis and unresolved issues of the role of *NOS1*, *NOS2*, *NOS3* genes as genetic predictors of the AH + TTH phenotype.

All three isoforms of the protein (nNOS, iNOS, eNOS) and its genes (*NOS1, NOS2, NOS3)* described above play key roles in the pathogenesis of both AH and TTH. They also negatively influence the leading environmental trigger of these nosologies: stress (Figure 2) and related neuropsychological disorders (anxiety and depression) [75].

**Figure 3:**
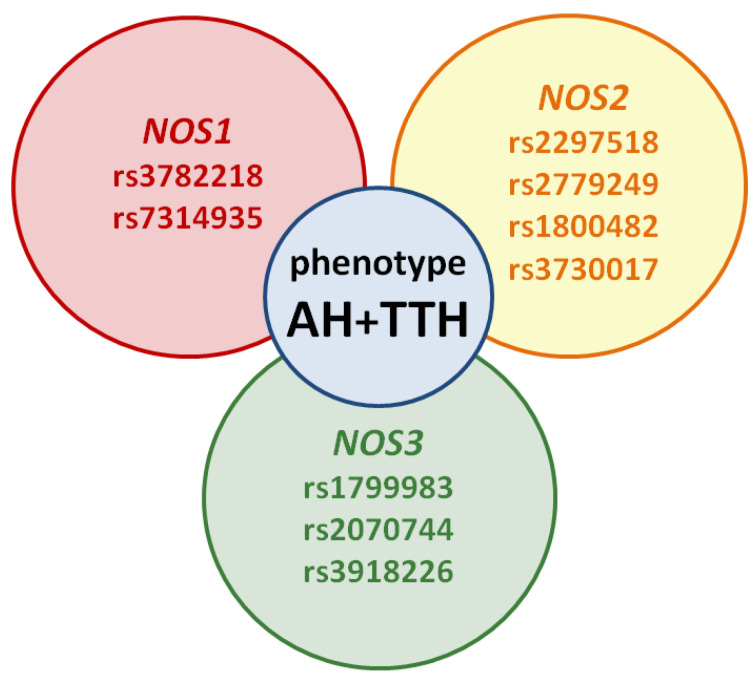
Potential SNVs of *NOS1*, *NOS2*, *NOS3* genes predisposed to the AH + TTH phenotype.

However, the prognostic role of *NOS1*, *NOS2*, *NOS3* genes in the development of the common AH + TTH phenotype has not been studied in comparison with the second most common phenotype (AH + migraine). Our analysis testifies to the importance of planning and conducting associative genetic research in the role of *NOS1*, *NOS2*, *NOS3* genes as genetic predictors of the AH + TTH phenotype (Figure 3) in various racial and ethnic groups.

This is important from a scientific and clinical point of view, because a new class of drugs that inhibit NOSs has been proposed in recent years, both for the treatment of AH and TTH. A new strategy for predicting and disease-modifying therapy of the common AH + TTH phenotype can increase the effectiveness and safety of treatment, improve patient’s quality of life, and reduce the risk of life-threatening cardiovascular complications.

## 4. Materials and Methods

We searched for full-text English publications from the past 15 years in the PubMed, Springer, Scopus, Web of Science, Clinicalkeys, and Google Scholar databases using keywords and combined word searches (nitric oxide; nitric oxide synthase; single nucleotide variants; single nucleotide polymorphisms; genes; *NOS1; NOS2*; *NOS3*; arterial hypertension; tension-type headache). We considered studies published from 2005 to 2020 and identified 32 publications devoted to the search for genetic predictors of the NO-synthase system in the development of AH and headache. Based on search criteria, only 24 of these publications were included in this review. In our review, we summed up SNVs of *NOS1*, *NOS2*, *NOS3* genes involved in the development of essential AH and headache. In addition, three earlier publications were included (1998, 2001 and 2003), to compare more racial and ethnic groups. The results of studies we have discussed in this review are contradictory. This might be due to different designs of the studies, small sample sizes in some of them, as well as different social and geographical characteristics.

## 5. Conclusions

NO has a free electron (NO), but despite being a free radical, it is not toxic as such. By reacting with superoxide, however, it forms peroxynitrite, which is a highly reactive free radical that exerts noxious effects on tissues. This mechanism is used in defense against infections, when iNOS is activated and produces NO in high concentrations. It is uncertain whether free radical formation plays a role at the lower concentrations of NO seen in endothelium and neurons [76].

The results of studies we have discussed in this review are contradictory, which might be due to different designs of the studies, small sample sizes in some of them, as well as different social and geographical characteristics. However, the contribution of genetic and environmental factors has been understudied, which makes this issue interesting for researchers, as understanding these mechanisms can support a search for new approaches to pathogenetic and disease-modifying treatment of the AH and TTH phenotype. New drugs against AH and TTH can be based on inhibition of nitric oxide (NO) production, blockade of steps in the NO-cGMP pathway, or NO scavenging [76].
